# Bat-Associated Parvoviruses: High Genetic Diversity and Novel Viruses in Gia Lai and Dong Nai Provinces, Vietnam

**DOI:** 10.3390/v18080860

**Published:** 2026-08-06

**Authors:** Vasilina K. Lapshina, Alina A. Gorbacheva, Polina A. Nikolaeva, Diana A. Grigoryan, Ivan F. Stetsenko, Mo T. Luong, Truong V. Tran, Alexander P. Yuzefovich, Ilia O. Prikhodko, Alexander Yu. Alekseev, Sergey M. Yudin, German A. Shipulin, Alina D. Matsvay, Veronika I. Skvortsova

**Affiliations:** 1 Federal State Budgetary Institution «Centre for Strategic Planning and Management of Biomedical Health Risks» of the Federal Medical Biological Agency, Moscow 119121, Russia; 2 Southern Branch of Joint Vietnam-Russia Tropical Science and Technology Research Center, Ho Chi Minh City 740500, Vietnamtruongleky@gmail.com (T.V.T.); 3 Research Institute of Virology, Federal Research Center of Fundamental and Translational Medicine, Novosibirsk 630090, Russia; 4 Department of Vertebrate Zoology, Moscow M.V. Lomonosov State University, Moscow 119234, Russia; 5 Federal Medical Biological Agency, Moscow 123182, Russia

**Keywords:** chiroptera, bats, virome, parvoviridae, parvovirus, Vietnam, metagenomics

## Abstract

Bats are recognized as key reservoirs for diverse viruses, including members of the *Parvoviridae* family. Vietnam hosts an exceptionally wide variety of bat species, yet the diversity of parvoviruses in these populations remains largely unexplored. This study provides the first survey of parvoviruses in bats from Gia Lai and Dong Nai provinces, screening 150 samples from 26 bat species and detecting parvoviruses in 38 (25%). A total of 45 viral operational taxonomic units (OTUs) were identified, encompassing four parvoviral subfamilies: *Parvovirinae*, *Densovirinae*, *Penbrevirinae*, and *Hamavirinae*. However, only two of these OTUs were attributed to ICTV-accepted species. Twenty-six OTUs were assigned to recognized genera but exhibited insufficient sequence similarity to established species, suggesting they may represent novel species within these genera. The remaining 25 OTUs could not be assigned to any ICTV-accepted genus, including 14 that remained unclassified even at the subfamily level, indicating the presence of novel parvovirus lineages at the genus rank or above. Overall, 95% of detected viral variants were classified as potentially novel, revealing a substantial reservoir of *Parvoviridae* diversity in the region. Phylogenetic analysis of vertebrate-infecting parvoviruses showed that the detected dependoparvoviruses were most closely related to viruses from primates and pinnipeds, while the chaphamavirus sequences formed a clade associated with carnivore viruses, and the embehamavirus sequences showed high homology to a virus previously detected in human plasma from a neuroinfection case. These results underscore the importance of comprehensive bat virome surveillance and enhance our understanding of the viral diversity and potential zoonotic threats posed by parvoviruses.

## 1. Introduction

The *Parvoviridae* family comprises small, non-enveloped, single-stranded DNA viruses renowned for their environmental persistence and prolonged infectious potential [1]. Parvoviruses rely on host cell replication machinery due to their limited coding capacity, which contributes to their narrow tissue and species tropism. Members of the genus *Dependoparvovirus* additionally require co-infection with a helper viruses to facilitate their replication cycle [1,2].

Taxonomically, the family was long classified into two well-established subfamilies: *Densovirinae*, which infects invertebrates, and *Parvovirinae*, pathogenic to vertebrates [1]. A third subfamily, *Hamaparvovirinae*, was proposed in 2019 [3]. These viruses exhibit unique genomic features intermediate between vertebrate and invertebrate parvoviruses. Subsequent International Committee on Taxonomy of Viruses (ICTV) taxonomic revisions replaced *Hamaparvovirinae* with the subfamilies *Hamavirinae*, *Hepanvirinae*, and *Penbrevirinae* and established the additional subfamily *Metallovirinae*, reflecting the expanding taxonomic diversity of the family [4].

Within *Parvovirinae*, eight monophyletic genera are recognized, five of which include human pathogens. While most associated infections are not life-threatening [1,5], several viruses are of clinical importance. Parvovirus B19, one of the first identified members, is linked to a spectrum of hematological and dermatological disorders [6] and human bocavirus 1, which is associated with respiratory disease. Other identified human parvoviruses, such as human bocavirus 2–4 [7,8,9], parvovirus 4 [10] and bufavirus [11], have been detected in clinical specimens, but their full clinical significance remains to be determined [5]. This stands in contrast to viruses infecting carnivores, such as canine parvovirus. Canine parvovirus emerged via interspecies transmission from feline populations, leading to a global pandemic in dogs with a reported mortality rate of up to 90% in untreated cases [12,13].

The next-generation sequencing has dramatically accelerated the discovery of novel *Parvoviridae* members and the elucidation of their evolutionary pathways. For instance, high-throughput sequencing has facilitated the identification of previously unknown parvoviruses, including chappaparvoviruses in dogs [14] and densoviruses in ticks [15]. Bats are recognized as a major natural reservoir for diverse viruses; however, the full extent of parvovirus diversity within bat populations remains largely unexplored [16]. Existing data indicate significant viral richness in this host group, with evidence of interspecies transmission, such as bocaparvovirus exchange between bats and pigs, as well as active intraspecies circulation [17,18].

The territory of Vietnam hosts a rich diversity of bat species, attributed to its complex topography and position within major biogeographic transition zones [19,20,21]. Key regions, such as the Tay Nguyen Plateau, act as crucial dispersal corridors where the faunas of Northern and Southern Indochina converge [19,20,21,22]. This ecological setting fosters the formation of complex viral communities and elevates the risk of zoonotic spillover, underscoring the critical need for targeted virome studies in local bat populations [23,24].

Despite the documented diversity of bat-associated parvoviruses in neighboring China [25,26], systematic investigations of parvoviruses circulating in Vietnamese bats remain lacking. Filling this surveillance gap is essential for understanding viral ecology and assessing potential public health threats in this biodiverse region.

To enhance the efficiency of virological surveillance, this study used targeted PCR followed by high-throughput sequencing for the directed screening and identification of a broad spectrum of members within the subfamily *Parvovirinae*. This work represents the first comprehensive assessment of parvoviruses detected in bats of Vietnam. This study focuses on populations from two key regions—Gia Lai and Dong Nai provinces—and aims to assess the prevalence and genetic diversity of parvoviruses in Vietnamese bats. This constitutes a step toward understanding their evolution and evaluating potential zoonotic transmission risks in the region.

## 2. Materials and Methods

### 2.1. Sample Collection and Preparation

Individual bats were captured using mist nets at two distinct sites: Kon Chu Rang Nature Reserve in Gia Lai Province (*n* = 63 samples) and Bu Gia Map National Park in Dong Nai Province (*n* = 87 samples) from 22 May to 3 June 2025. A total of 150 individual bat fecal samples were collected. After releasing from the net, each bat was temporarily placed in an individual clean cloth bag, and a single fecal sample was collected from each individual. Following sample collection, all bats were released at the site of capture.

Mist net capture was performed only once per location to minimize the chance of duplicate sampling. Field procedures included species identification using a standard taxonomic guide [21]. All handling was performed by trained personnel in accordance with ethical standards for wildlife research, under a protocol approved by the local ethics committee of the Joint Russian-Vietnamese Scientific Research and Technology Center (Protocol No. 1824 dated 21 May 2025-approval number 1837/CN-HĐĐĐĐV dated 21 May 2025).

Each sample was immediately placed into an individual 2.0 mL cryovial containing phosphate-buffered saline (PBS). Samples were flash-frozen in liquid nitrogen in the field and subsequently transferred to the laboratory for long-term storage at −80 °C.

### 2.2. Total Nucleic Acid Extraction

Each fecal sample was homogenized by vortexing for a minimum of two minutes. Homogenization was assessed visually based on the uniformity of particle distribution and sample consistency. The homogenate was then subjected to two cycles of sedimentation to remove large particulate debris. Following each cycle, the clarified supernatant was collected after centrifugation at 13,000× *g* for 1–2 min.

Total nucleic acid was extracted from 100 µL of the clarified homogenate for each sample using the RIBO-prep kit (AmpliTest, Moscow, Russia) according to the manufacturer’s instructions. The purified nucleic acids were eluted and stored in LowTE buffer. The collected bat samples were analyzed using both targeted and metagenomic sequencing approaches.

### 2.3. Targeted PCR Amplification, Sequencing, and Raw Data Processing

For targeted sequencing, the samples were screened using a pool of specific primers (Table 1). The primer set was designed based on a database comprising 760 complete genomic sequences of mammal-associated parvoviruses, retrieved from NCBI GenBank [27]. The exact composition of the database used for primer design is provided in Supplementary Table S3. To evaluate primer coverage in silico, local alignments were constructed against the reference database. The designed primer panel covered 99% of the target sequences, with no mismatches permitted within the terminal five bases at the 3′ end and a maximum of one mismatch across the full primer length alignment.

Primers were used at a final concentration of 1 µM of each. In each reaction, 5 µL of isolated total nucleic acid served as the template. Amplification was performed in a 25 µL reaction mixture containing TaqF polymerase (AmpliTest, Moscow, Russia) under the following conditions: an initial denaturation at 95 °C for 15 min, followed by 45 cycles of 95 °C for 30 s, 50 °C for 90 s, and 72 °C for 30 s, with a final extension at 72 °C for 5 min. The resulting amplicons were purified using MagPure A4 XP magnetic beads (Magen, Guangzhou, China).

Sequencing libraries were prepared according to a previously published protocol [28] and sequenced on a portable sequencer using an R10.4.1 flow cell (Oxford Nanopore Technologies, Oxford, UK), with read counts per sample ranging from 3297 to 657,370 and an average of approximately 219,000 reads. Bioinformatics analysis for the targeted data was conducted using the PathogenID software v 1.3.2 [29], which provides an integrated pipeline for processing nanopore data from raw reads to a final report detailing the taxonomic classification of identified genomic sequences. The obtained amplicon sequences ranged in length from 145 to 1438 bp.

### 2.4. Metagenomic Library Preparation, Sequencing, and Raw Data Processing

For metagenomic sequencing, nucleic acids were purified using MagPure A4 XP magnetic beads (Magen, Guangzhou, China) at a 1.2:1 bead-to-sample ratio. Libraries were prepared using the NEBNext Ultra™ II RNA Library Prep Kit (New England Biolabs, Ipswich, MA, USA) in combination with adapters and PCR barcodes from the PCR Barcoding Expansion 1-96 (Oxford Nanopore Technologies, Oxford, UK). Motor protein ligation was performed according to the manufacturer’s standard protocol. Sequencing were conducted on a portable sequencer using an R10.4.1 flow cell (Oxford Nanopore Technologies, Oxford, UK).

Bioinformatic processing of metagenomic sequences involved several sequential steps. Raw read basecalling was performed with Dorado v.7.2.13, after which adapters were trimmed using Porechop v.0.2.4 [30].

### 2.5. Phylogenetic Analysis

For phylogenetic analysis of the viruses, the following loci were used. For *Dependoparvovirus*, the replication protein locus (NS1) was used, resulting in an alignment of 826 positions. For *Bocaparvovirus* and *Hamavirinae*, analysis was performed using the capsid protein, with alignment lengths of 751 and 805 amino acids, respectively. The choice of genomic region for phylogenetic analysis was determined by the availability of loci in the obtained sequences. The phylogenetic tree topology obtained for the VP protein was consistent with that derived from theNS1 using the ICTV reference set [4], supporting the taxonomic classification established by the International Committee on Taxonomy of Viruses [4,31]. A broad-scale analysis to position novel viruses within the *Parvoviridae* was based on a dataset consisted of a single representative of each *Parvoviridae* genus, with *Caliciviridae* used as outgroup. The final dataset included 39 sequences.

Multiple sequence alignment for all genera was performed with MAFFT v.7.525 [32] using a reference dataset based on the ICTV species list [4]. The alignments were trimmed using the clipkit v2.7.0 program (in ‘--smartgap’ mode) [33]. An alignment of the helicase domain (SF3 helicase) of the NS1 protein, made with T-Coffee [34], was analyzed under the LG + I + G4 model with a lognormal relaxed clock and Yule speciation model for 100 million generations. Genetic distances (amino acid differences per site) were calculated in MEGA11 [35] using a gamma-distributed rate variation.

All ML phylogenetic trees were constructed using RAxML-NG v. 1.2.2 under the LG + I + G4 + F model with 1000 bootstrap replicates [36]. The optimal evolutionary model was selected using ModelTest-NG v0.1.7 [37]. Tangelgram plots was built with the usage of RStudio v4.4.2 [38] using libraries ape [39], phytools [40], and dendextend [41]. The comparison of VP1/VP2 and NS1-based trees is shown in Supplementary Figures S1–S3.

### 2.6. Genome Assembly, Annotation, and Homologue Analysis

Contigs were generated through de novo assembly using Canu v.2.2 [42]. Non-viral sequences, particularly bacterial reads, were eliminated with Kraken2 v2.1.3 [43]. A hybrid taxonomic identification approach was applied, combining BLASTn v2.16.0+ [44] for nucleotide queries and DIAMOND v2.1.16 [45] for protein homology searches, facilitating the recognition of both familiar and highly divergent viral sequences. The assemblies underwent final refinement with Pilon v1.24 [46] to correct residual errors.

Genome annotation and visualization were performed manually using SnapGene Viewer v6.2.1 [47].

Coverage and identity scores were calculated with BLAST v2.16.0+ [44], results has been visualized with RStudio v4.4.2 using ggplot2 library [48].

### 2.7. Statistical and Correlation Analysis

To identify significant associations between viral groups and ecological factors (geographic regions and host bat diet), adjusted standardized residuals (AR) were calculated. The strength of these associations was assessed using Pearson’s coefficient of contingency (C). All statistical analyses were performed using PAST software v 4.13 [49].

## 3. Results

### 3.1. Taxonomic Diversity of the Sampled Bats

**Figure 1:**
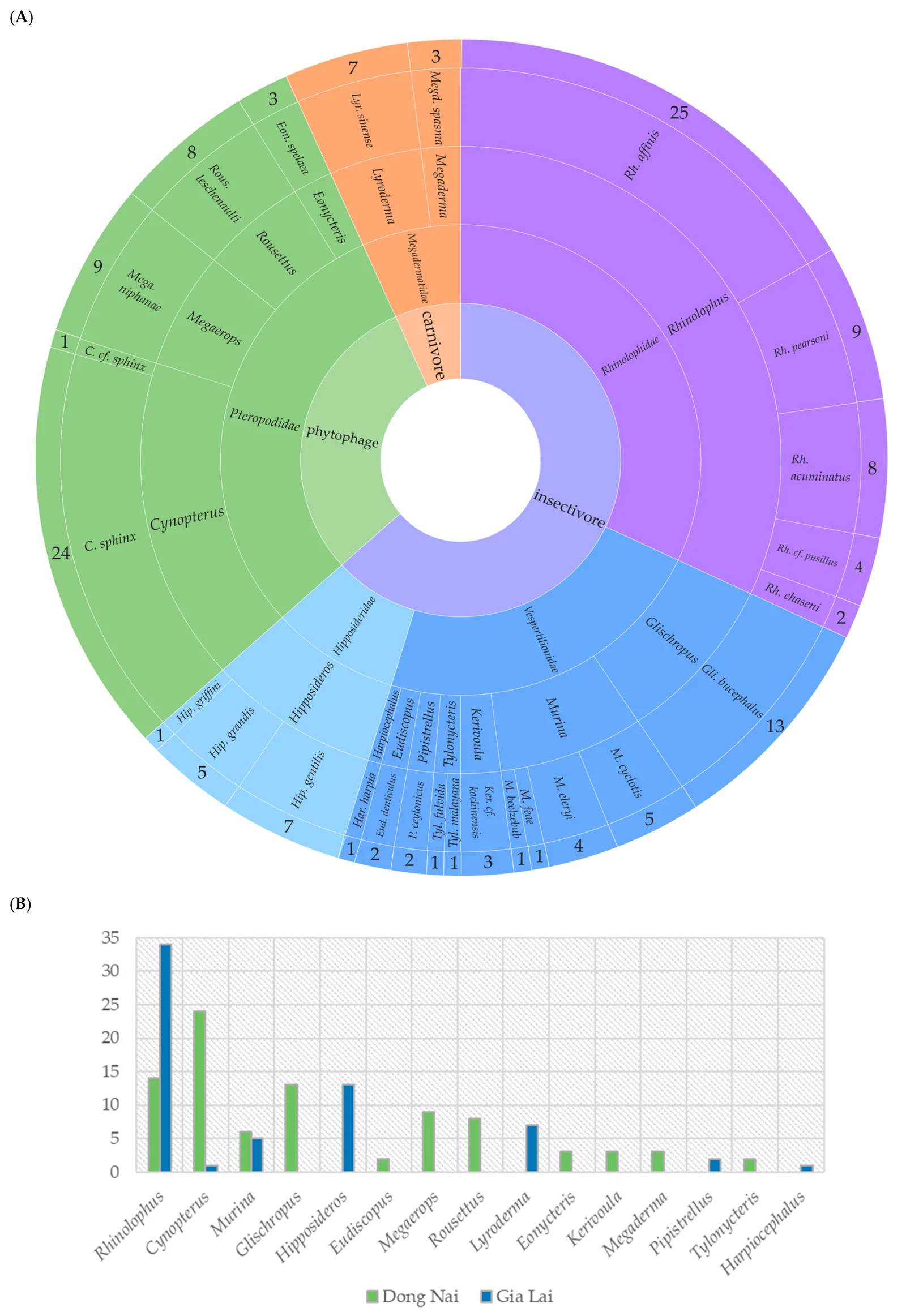

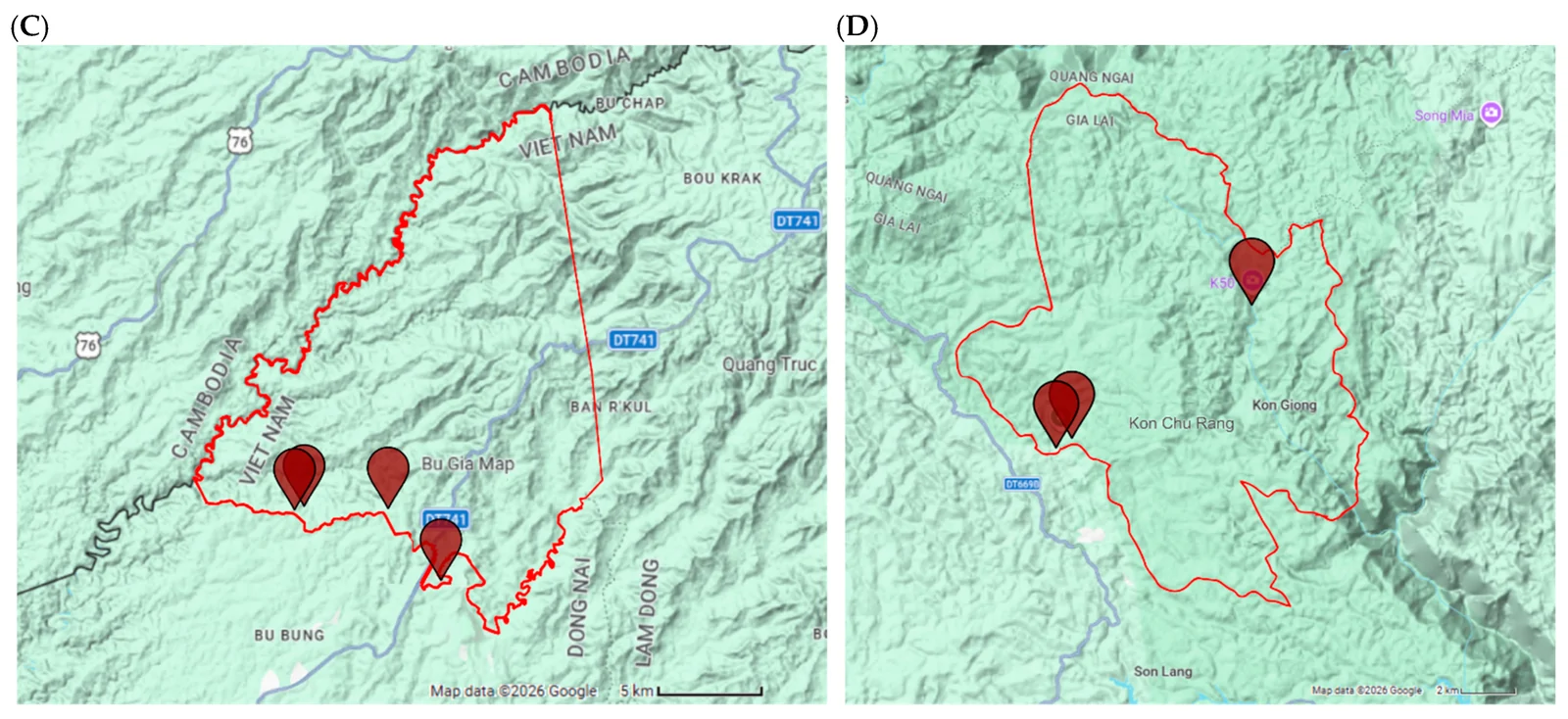
(**A**) This hierarchical sunburst diagram visualizes the structure and relative abundance of 150 individual bats from fecal samples collected in Gia Lai and Dong Nai provinces. The concentric rings represent, from innermost to outermost, bat family, genus, and species. The length of each segment corresponds to the number of individuals identified for that taxonomic rank. The center ring color-codes the primary dietary specialization (phytophages, insectivore, and carnivore) for each corresponding species. (**B**) Comparative bat genus representation in two Vietnamese provinces: Dong Nai (green) and Gia Lai (blue). The vertical axis (*y*-axis) shows the number of samples; the horizontal axis (*x*-axis) lists 15 identified bat genera. (**C**) Map showing sampling sites within Bu Gia Map National Park, Dong Nai Province, and (**D**) Kon Chu Rang Nature Reserve, Gia Lai Province.

During field research in two provinces of Vietnam, the 150 individual fecal samples were collected from a taxonomically diverse cohort of bats, encompassing 5 families, 15 genera, and 26 species (Figure 1). The most abundant species were *Rhinolophus affinis* (25 individuals) and *Cynopterus sphinx* (24 individuals). *C. sphinx* was the only species detected at both sampling locations (Gia Lai and Dong Nai provinces). This species is widely distributed throughout Southeast Asia and is commonly associated with anthropogenic and disturbed landscapes [21].Three bat genera, *Cynopterus*, *Murina*, and *Rhinolophus*, were common to both provinces. In Dong Nai Province, 16 bat species were identified, mirroring the overall sample diversity. The common short-nosed fruit bat (*C. sphinx*) was the dominant species, represented by 23 individuals. In contrast, 11 species were recorded in Gia Lai Province, where the dominant species was *Rh. affinis*. Despite differences in species composition, the majority of species in both provinces were insectivorous. A more detailed analysis of the trophic structure and its potential relationship with viral infection patterns will be presented in the subsequent section.

### 3.2. Genetic Diversity of Parvoviruses

**Figure 2:**
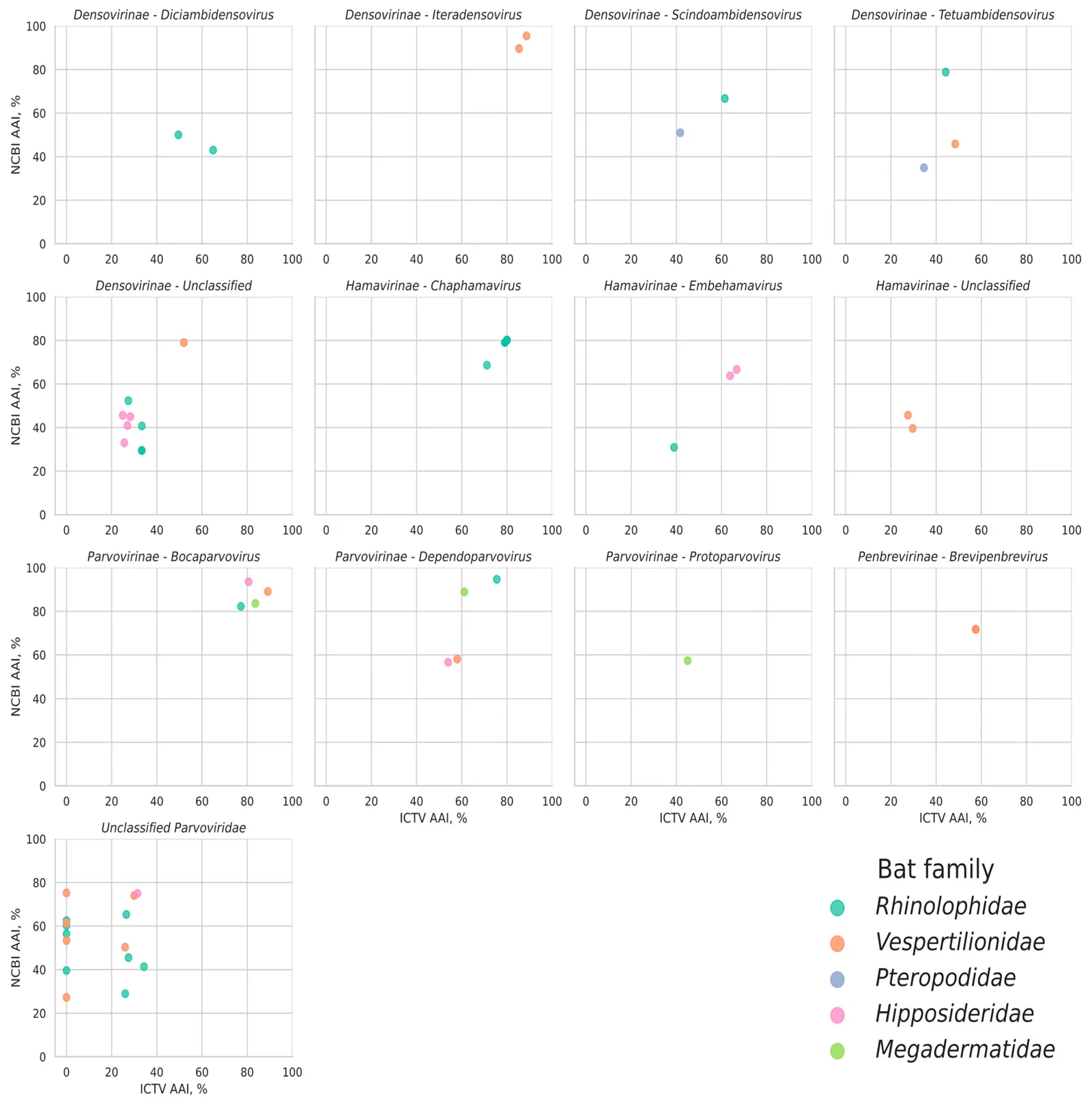
Distribution of amino acid identity (AAI) for bat-associated parvoviruses relative to ICTV reference sequences and NCBI GenBank database. The figure consists of 13 panels, each corresponding to a genus within the family Parvoviridae or shows unclassified sequences. In each panel, individual viral sequences are represented as dots. The horizontal axis (*X*-axis) shows the percentage of amino acid identity (AAI, %) to the best-matching reference sequence from the officially approved ICTV species. The vertical axis (*Y*-axis) displays the AAI (%) to the top hit in the NCBI GenBank database. Points are color-coded according to the host bat family from which the parvovirus sequence was obtained, as indicated in the legend.

A total of 150 samples were screened for viral DNA. Information on all sampled bats, including sampling locations and parvovirus detection status, is provided in Supplementary (Table S1). Parvoviral DNA was detected in 38 samples (25.3% of the total). Reference sequences of species officially recognized by the ICTV were used for taxonomic assignment [4]. Formal taxonomic classification by the ICTV requires a complete coding genome, and species demarcation for parvoviruses is defined using NS1 protein identity. However, the sequences obtained in this study were predominantly partial, precluding formal classification. For samples lacking a suitable NS1 sequence, the VP locus was used as an alternative marker (Supplementary Table S2). Accordingly, ICTV thresholds were employed as a reference guide, enabling provisional taxonomic inference from the available partial sequences rather than definitive assignment. Amino acid identity (AAI) cutoffs of 85% and 30% were applied for species and genus assignments, respectively, with a minimum coverage threshold of 80% (Figure 2). Sequences without a consistent subfamily affiliation were assigned only to the family level (*Parvoviridae*) based on BLASTp v2.16.0+ searches against the NCBI non-redundant database. Thus, in this study, operational taxonomic units (OTUs) were defined as clusters of sequences sharing more than 85% pairwise AAI, in accordance with the ICTV species demarcation criterion. If different genomic regions were sequenced, this approach yields locus-specific OTU groups, which can inflate the total number of OTUs observed. All ls-OTUs were manually inspected, and those aligning to the same viral genome with similar identity percentages were merged.

Subsequent analysis identified 54 distinct viral sequences, which were grouped into 45 OTUs beloning to 10 genera distributed across four recognized subfamilies within the family *Parvoviridae* (*Parvovirinae*, *Densovirinae*, *Hamavirinae*, and *Penbrevirinae*), as well as multiple unclassified and transitional clades that currently lack formal taxonomic status. Only three of the detected parvovirus sequences (4%, 2 OTUs) met the species criterion, corresponding to *Bocaparvovirus chiropteran1* and *Iteradensovirus lepidopteran*. Detailed information is provided in Supplementary Materials (Table S2).

For 25 sequences (19 OTUs; 42%), assignment could only be made to the genus level. These sequences exhibited divergence from all recognized species exceeding the species demarcation threshold, indicating they may represent novel species within established genera.

A further 11 sequences (10 OTUs; 22%), only be classified at the subfamily level, as their divergence precluded genus-level classification. Within this group, AAI to the closest classified relatives were consistently low, ranging from 25% to 52% AAI.

A substantial fraction (15 sequences; 14 OTUs; 31%) of the detected sequences had no significant match to ICTV reference sequences below the family level. To investigate their taxonomic relations, we performed a BLAST search against the NCBI database. Resultant identities, to the closest known sequences of parvoviruses, ranged from 27% to 75% AAI with 65–98% coverage, suggesting that a significant proportion of these OTUs likely represent novel species.

### 3.3. Subfamily Densovirinae

Overall, the subfamily *Densovirinae* accounted for 35% of the total viral sequences identified. This distribution suggests a substantial presence of invertebrate-associated viruses, potentially reflecting the influence of diet or symbiotic arthropod hosts on the composition of the bat virome. Sequences classified as likely belonging to *Densovirinae* are of limited relevance for interpreting direct bat–virus relationships, as the primary hosts for members of this subfamily are invertebrates. Their detection in bat fecal samples likely reflects dietary exposure to infected arthropods rather than active infection of the bats themselves. This distinction is important when interpreting host–virus relationships in metagenomic studies, as it may indicate widespread circulation of densoviruses in local arthropod populations rather than in bats. Consequently, although detection of these sequences does not provide well-supported information about bat virome, it results in insights into the broader virus diversity of tropical ecosystems in Vietnam.

### 3.4. Subfamily Parvovirinae

Viral sequences of the *Parvovirinae* subfamily was identified in eight samples, accounted for nine genomic sequences (eight OTUs), representing 17% of the total dataset. The detected sequences were attributed to the genera *Bocaparvovirus*, *Dependoparvovirus*, and *Protoparvovirus*, with the majority belonging to the former two.

#### 3.4.1. *Bocaparvovirus* Genus

**Figure 3:**
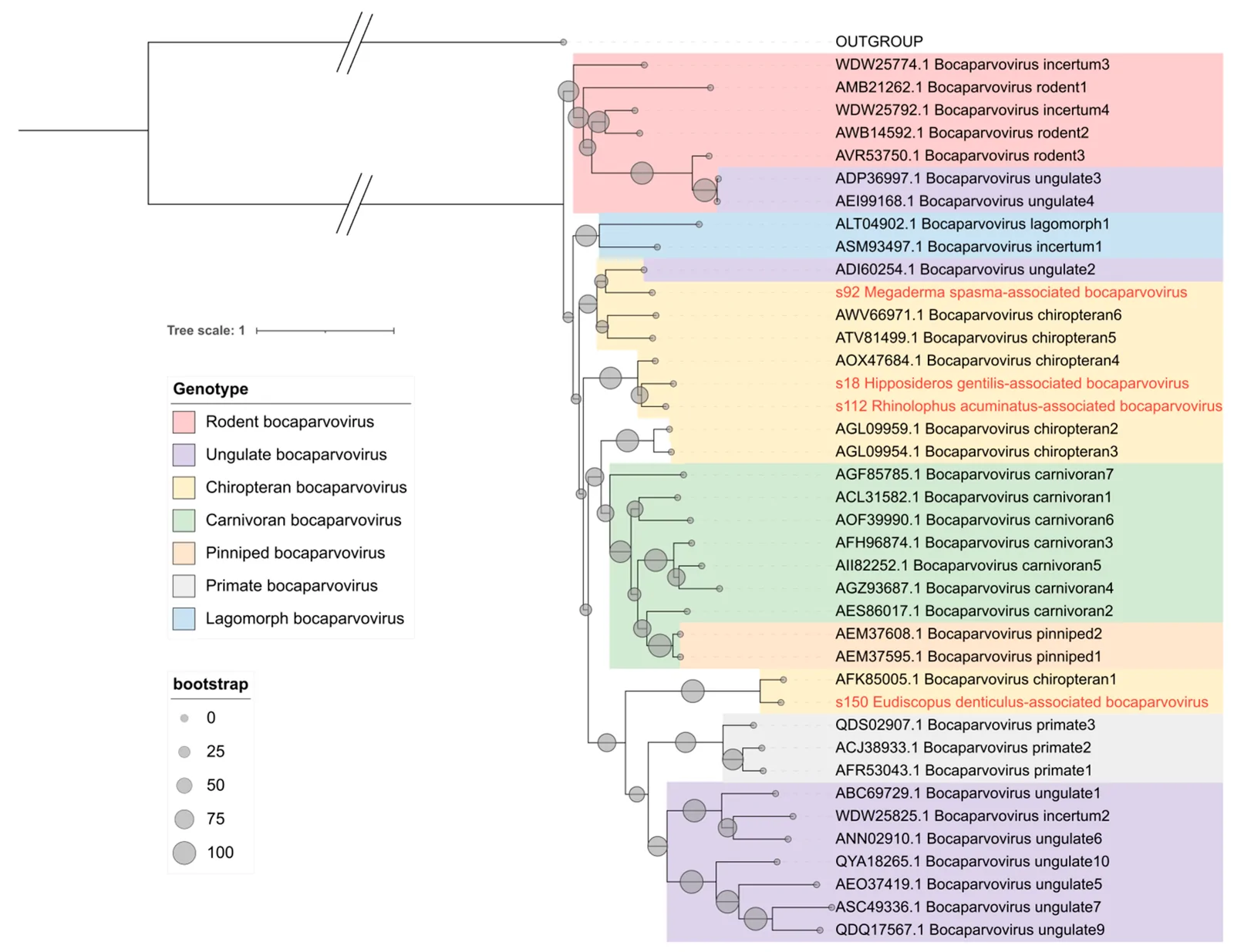
Phylogenetic analysis of *Bocaparvovirus* VP1 capsid protein amino acid sequences. The maximum-likelihood tree depicts the evolutionary relationships among *Bocaparvovirus* sequences from the ICTV reference set. Major clades, defined by host association (e.g., chiropteran and carnivoran), are distinguished by colored backgrounds and labeled in the accompanying legend. Sequences identified in this study are highlighted in red. Bootstrap support values (from 1000 replicates) are shown at key nodes.

Among the Bat-specific bocaparvoviruses, four sequences (four OTUs) homologous to *Bocaparvovirus chiropteran1/4/5* were identified; AAI values ranged from 77 to 89% at the amino acid level (95–98% coverage), confirming their placement within this group. Phylogenetic analysis of VP1 capsid protein amino acid sequences places all *Bocaparvovirus* sequences from this study within established clades of chiropteran bocaparvoviruses (Figure 3). This finding aligns with the broader diversity of previously reported bat bocaparvoviruses, which are genetically heterogeneous and form several distant, paraphyletic groups.

Our analysis revealed several distinct phylogenetic patterns among the newly identified bocaparvoviruses. One sequence, classified as belonging to *Bocaparvovirus chiropteran 1* and isolated from the insectivorous bat *Eudiscopus denticulus* in Dong Nai Province (s150), showed a high degree of similarity (89% AAI, 95% coverage) to known references and formed a distinct and divergent clade separate from other chiropteran bocaparvoviruses together with Bocaparvovirus chiropteran1 (AFK85005.1).

A second well-supported monophyletic group consists of two sequences (2 OTUs) obtained from *Hipposideros gentilis* (s18) and *Rh. acuminatus* (s112). These sequences form a highly supported clade with their closest known relative, *Bocaparvovirus chiropteran 4* (AOX47684.1). However, given their considerable genetic and phylogenetic distance from this reference sequence (81% AAI for s18 and 77% AAI for s112 with 98% coverage for both) and from each other (78% AAI), both sequences should be considered as representatives of two novel bocaparvovirus species.

The third clade comprises reference sequences for *Bocaparvovirus chiropteran5* (ATV81499.1, phytophages, China) and *Bocaparvovirus chiropteran6* (AWV66971.1, phytophages, Cameroon), *Bocaparvovirus ungulate2* (ADI60254.1, Porcine, China), and a novel sequence from *Megaderma spasma* (facultative carnivore). The novel sequence is closely related to *Bocaparvovirus chiropteran5* (s 92, 84% AAI, 97% coverage), falling marginally below the species threshold.

#### 3.4.2. *Protoparvovirus* Genus

A single protoparvovirus sequence was identified, from the carnivorous bat *Lyroderma sinense* (s24), exhibiting low amino acid identity (45% AAI, 80% coverage) to its closest known relative, *Protoparvovirus chiropteran2*.

**Figure 4:**
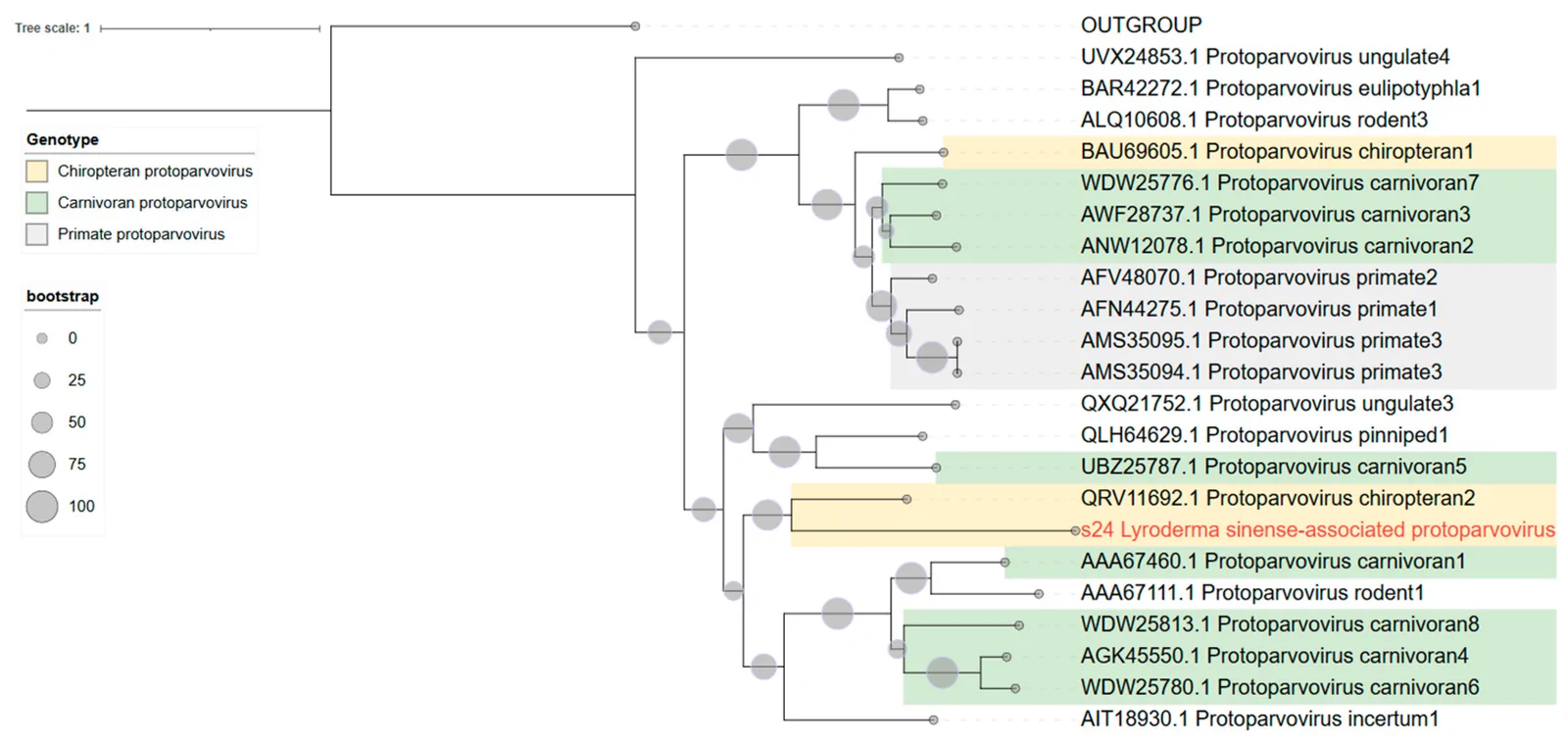
Phylogenetic analysis of Protoparvovirus VP1 gene sequences. The maximum-likelihood tree illustrates the evolutionary relationships among Protoparvovirus sequences from the ICTV reference set. Major clades, corresponding to host association (chiropteran, carnivoran, and primate), are indicated by colored backgrounds as defined in the legend. Sequences identified in this study are highlighted in red. Bootstrap support values (based on 1000 replicates) are shown at key nodes.

Phylogenetic analysis (Figure 4) placed it on a branch with *Protoparvovirus chiropteran2* (QRV11692.1) but at considerable evolutionary distance. Notably, the clade sister to this group comprised carnivoran-associated parvoviruses.

#### 3.4.3. *Dependoparvovirus* Genus

Analysis identified four sequences belonging to the genus *Dependoparvovirus*, all related to adeno-associated viruses (AAVs). The sequences were obtained from four individual bats sampled in Kon Chu Rang (Gia Lai Province) and represent four distinct OTUs; the best AAI with ICTV reference sequences ranged from 54 to 76% (94–98% coverage).

**Figure 5:**
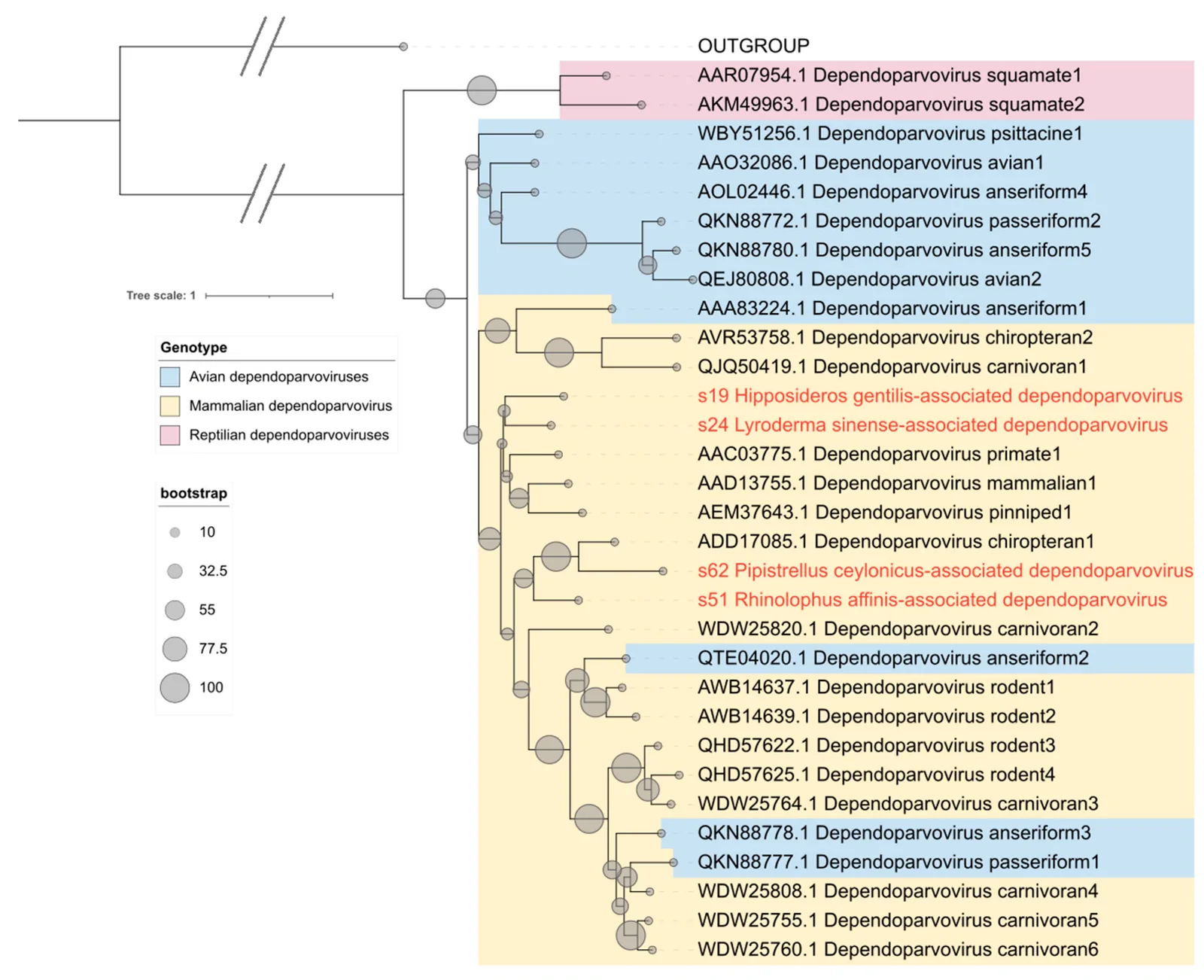
Phylogenetic analysis of *Dependoparvovirus* NS1 gene sequences. The maximum-likelihood tree illustrates the evolutionary relationships among *Dependoparvovirus* sequences from the ICTV reference set. Major clades, corresponding to host association (chiropteran, avian, and reptilian), are indicated by colored backgrounds as defined in the legend. Sequences identified in this study are highlighted in red. Bootstrap support values (based on 1000 replicates) are shown at key nodes.

Phylogenetic analysis (Figure 5) confirms three major host-associated clades (reptilian, avian, mammalian). Known chiropteran dependoparvoviruses form paraphyletic lineages within the mammalian clade, a pattern extended by our data.

Our viral sequences from *Lyroderma sinense* (s24) and *Hip. gentilis* (s19) formed a distinct branch most closely related to the primate dependoparvovirus clade, with best AAI of 61% (94% coverage) and 54% (98% coverage), respectively, to *Dependoparvovirus primate1*. Separately, sequences from *Pipistrellus ceylonicus* (s62) and *Rh. affinis* (s51) grouped with the *Dependoparvovirus chiropteran1* reference sequence, sharing 58% (97% coverage) and 76% AAI (96% coverage), respectively.

### 3.5. Subfamily Penbrevirinae

#### *Brevipenbrevirus* Genus

A single *Penbrevirinae* OTU was detected in Dong Nai Province, comprising two sequences from two individual *Glischropus bucephalus* bats. Both exhibited 58% AAI and 95% coverage to their closest database relative, *Brevipenbrevirus orthopteran2*. Given that the *Brevipenbrevirus* genus is primarily associated with insect hosts, the detected sequences may be linked to the bats’ insectivorous diet than to a direct infection of the bat host.

### 3.6. Subfamily Hamavirinae

**Figure 6:**
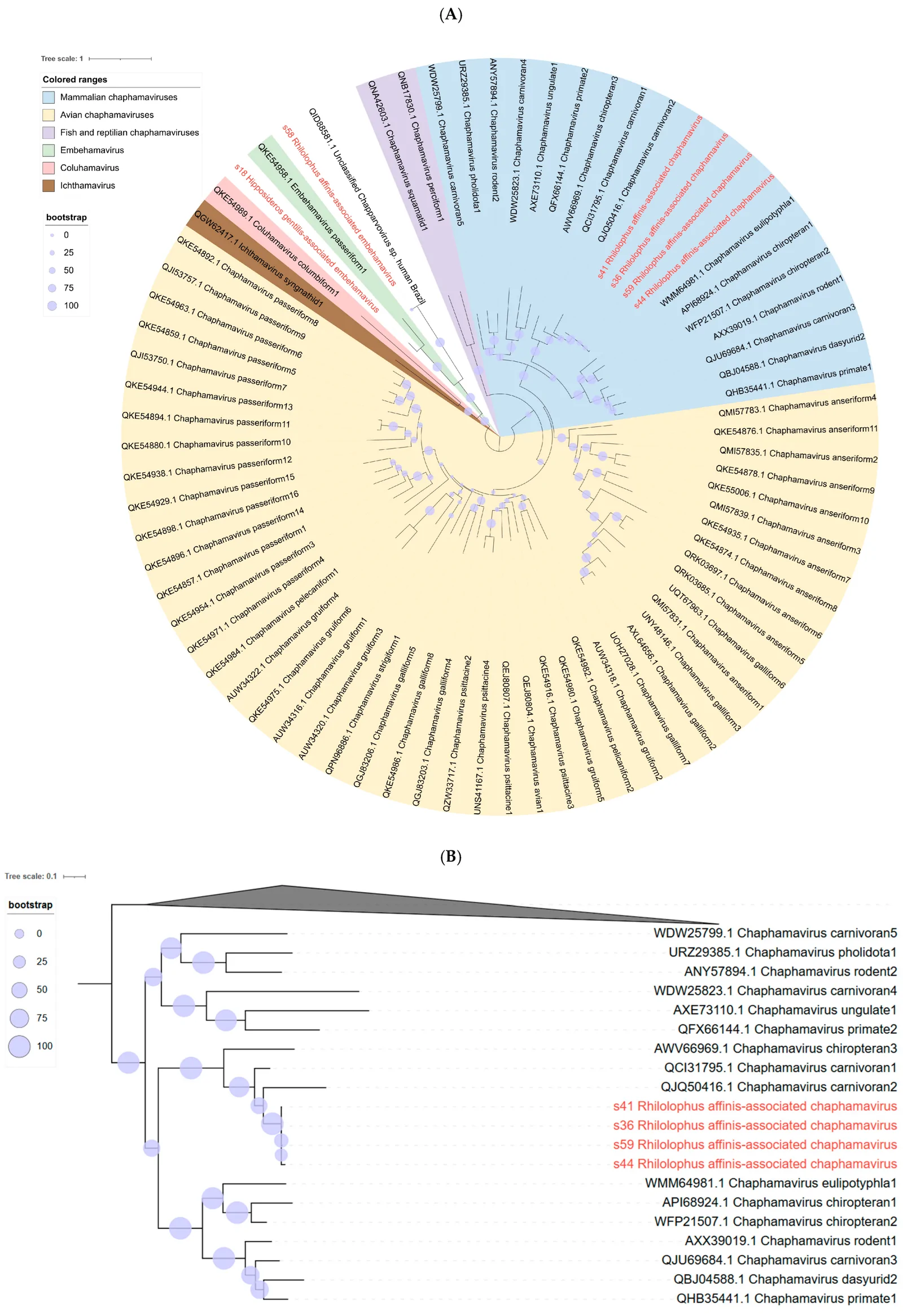

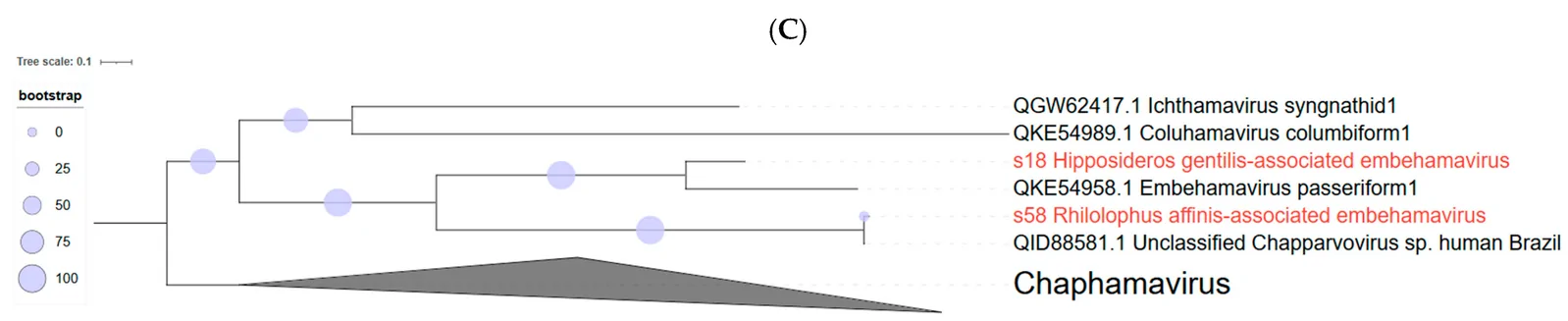
Phylogenetic analysis of *Hamavirinae* based on VP1 gene sequences. The maximum-likelihood tree was constructed using the ICTV reference set. Novel sequences identified in this study are highlighted in red. Bootstrap support values (from 1000 replicates) are indicated at key nodes. (**A**) Global phylogenetic tree showing major host-associated clades among *Hamavirinae* sequences from the ICTV reference set. Major clades corresponding to host association are indicated by colored backgrounds as defined in the legend. (**B**) Expanded view of the mammalian chaphamaparvovirus clade, which contains the sequences identified in this study. (**C**) Expanded views of clades comprising the genera Embhamaparvovirus and Ichthamaparvovirus, which contains the sequences identified in this study.

In our study, sequences belonging to the subfamily *Hamavirinae* were identified in both surveyed regions, comprising 18% (10 sequences) of the total viral sequences and representing six distinct OTUs. Four of these OTUs were classified as likely belonging to the genera *Embehamavirus* and *Chaphamavirus* (Figure 6), while the remaining two could not be classified at the genus level and showed low similarity to ICTV-recognized taxa. BLAST searches against the NCBI GenBank database identified all four sequences as most closely related to parvoviruses, including *Miniopterus bat parvovirus*, *Emberiza spodocephala parvoviridae* sp., *Chaerephon bat parvovirus*, and *Army ant associated chapparvovirus 13*, with AAI values ranging from 29% to 46% and coverage above 80% (Supplementary Materials, Table S2).

#### 3.6.1. *Chaphamavirus* Genus

One OTU was classified as likely belonging to the genus *Chaphamavirus*, which comprises vertebrate pathogens. This OTU comprised five sequences from *Rh. affinis*, all collected in Gia Lai Province, to *Chaphamavirus carnivoran1* with AAI of 71–79% for NS1 locus and 79–80% for VP1, coverage 96–100%. Based on the pairwise AAI values, this OTU may represent an previously undescribed species within the genus *Chaphamavirus*.

Phylogenetic analysis (Figure 6) showed that sequences corresponding to OTU closest to *Chaphamavirus carnivoran1* formed a well-supported clade that grouped with *C. carnivoran1* and *C. carnivoran2* (feline and canine origin, associated with diarrhea). This chiropteran clade is phylogenetically distinct from other bat-associated chaphamavirus lineages.

#### 3.6.2. *Embehamavirus* Genus

Another OTUs, associated with *Hip. gentilis* and *Rh. affinis*, formed a well-supported clade within the recently established genus *Embehamavirus*, which currently comprises a single officially recognized species, *Embehamavirus passeriform1*. The detected sequences shared 39–67% AAI, coverage above 80%, with the reference sequence, indicating genetic divergence from the currently recognized species of the genus.

Notably, the *R. affinis-associated embehamavirus* sequence (s58) shares 96% AAI with unclassified by ICTV *Chapparvovirus* sp. QID88581.1 was detected in a human plasma sample from Brazil, which, according to our phylogenetic reconstruction, falls within *Embehamavirus* clade. Despite lacking formal ICTV species status, QID88581 possesses a complete genome sequence and was included in our phylogenetic analysis because of its high similarity to the s58 sequence.

### 3.7. Unclassified Parvoviruses

The high proportion of sequences (15 sequences; 14 OTUs; 31%) could not be classified to the genus or even subfamily level. Sequences classifiable only at the family level (*Parvoviridae*) showed no significant similarity to any officially recognized ICTV species. BLASTp searches against the NCBI database revealed uniformly low AAI (27–75% with coverage 65–98%) to the closest GenBank sequences annotated as parvoviruses (Supplementary Materials, Table S2). Several of the GenBank matches, including *Emberiza pusilla parvoviridae* sp., *Luscinia sibilans parvoviridae* sp., and *Motacilla cinerea densovirus*, originated from studies describing highly divergent parvoviruses [50].

Several sequences exhibited closest matches to insect-associated viruses, including s39 (41% AAI with 97% coverage to *Army ant-associated bidensovirus 2*) and s48 (63% AAI with 73% coverage to *Parus major densovirus*) obtained from *Rh. affinis*, suggesting potential dietary exposure or unsampled viral diversity in invertebrate hosts. Notably, two sequences from *Pipistrellus ceylonicus* (s62) and *Hip. grandis* (s12) formed a shared OTU (OTU_4) with only 30–31% AAI to *Bocaparvovirus chiropteran5* (ICTV accepted) but 74–75% AAI (with 96% coverage) to a unclassified bat-associated parvovirus (*Parvovirus chalinolobus3580a*), indicating a chiropteran origin despite their distance from known ICTV species.

These ICTV-unclassified viruses therefore represent a complex of novel, genetically diverse taxa whose precise phylogenetic position within the *Parvoviridae* remains to be determined. Our findings highlight the existence of numerous divergent lineages in unexplored host systems and geographic regions, suggesting that current taxonomic frameworks capture only a fraction of the true parvovirus diversity circulating in nature.

### 3.8. Whole Genome Characterization of Novel Rhinolophus Affinis-Derived Parvovirus

**Figure 7:**
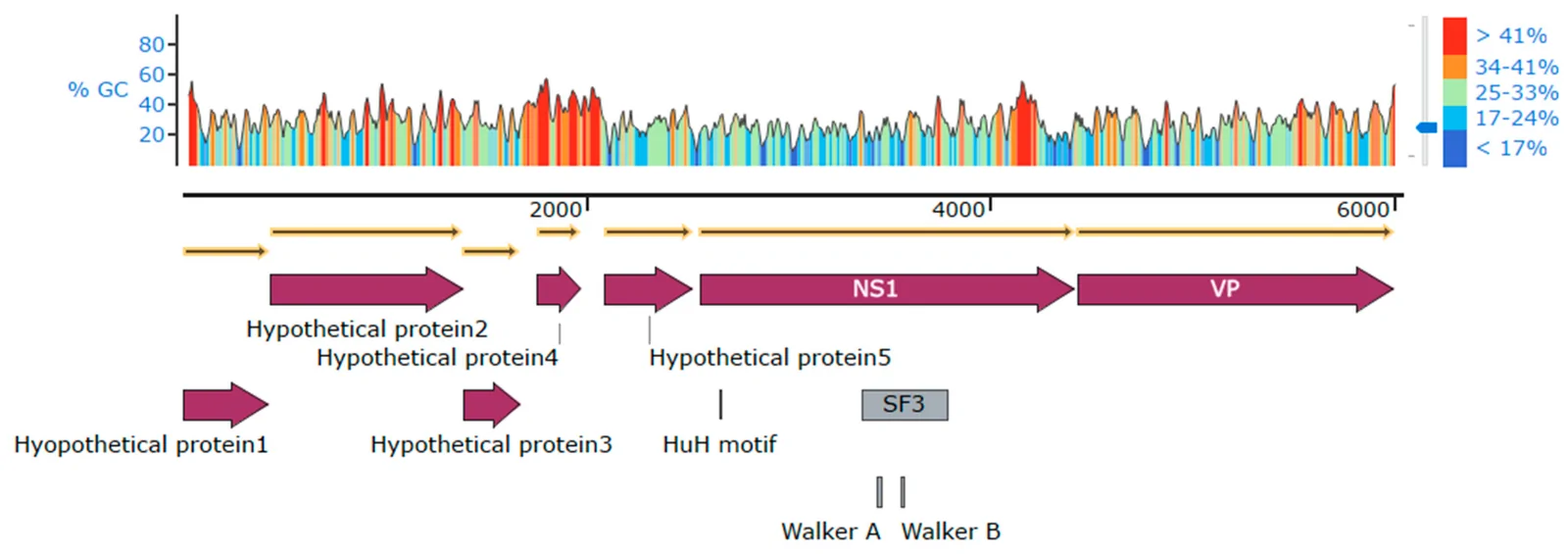
Genome organization of Rhinolophus affinis-derived parvovirus. The linear genomic map shows the predicted open reading frames (ORFs) (orange). The nonstructural protein (NS1) ORF is depicted in blue, and the capsid protein (VP) ORF is shown in purple. Conserved functional motifs within the NS1 protein are annotated: the superfamily 3 (SF3) helicase domain and the Walker A/B loops are highlighted in gray. The diagram was generated using SnapGene.

Genome sequencing and assembly yielded a near-complete genome of a novel virus (Figure 7), designated as *Rhinolophus affinis*-derived parvovirus (GenBank accession: PZ507329). The genome is 6036 bp in length, which falls within the ICTV-defined size range for the family *Parvoviridae* (4–6 kb). Both major open reading frames (ORFs) encoding the nonstructural protein NS1 (1869 bp, 622 amino acids) and the capsid protein VP (1578 bp, 525 amino acids) are present and correspond to expected lengths (Table 2). The NS1 protein contains a superfamily 3 (SF3) helicase domain (residues 368–510), including the Walker A motif (GxxxxGKT/S, GCPNAGKS), Walker B motif (xxxxEE, CALWEE), and the HuH endonuclease motif (HLH, residues 133–135). The VP protein lacks a detectable phospholipase A2 (PLA2) domain [31,51]. The absence of identifiable inverted terminal repeats (ITRs) is likely attributable to technical limitations of the sequencing and assembly methods used.

The genome also contains five shorter ORFs in the 5′ region, encoding hypothetical proteins of unknown function with no detectable similarity to any sequences in NCBI GenBank. Structural predictions using AlphaFold yielded pLDDT scores ranging from 34.25 to 39.89, indicating low confidence. Given the absence of homologous sequences, definitive functional characterization of these hypothetical proteins is not possible without further investigation.

Based on its structural and functional features, including the presence of both NS1 and VP ORFs, a genome length within the established range, and the presence of the SF3 helicase domain in NS1, this virus fulfills the primary ICTV criteria for classification within the family *Parvoviridae* [1].

**Figure 8:**
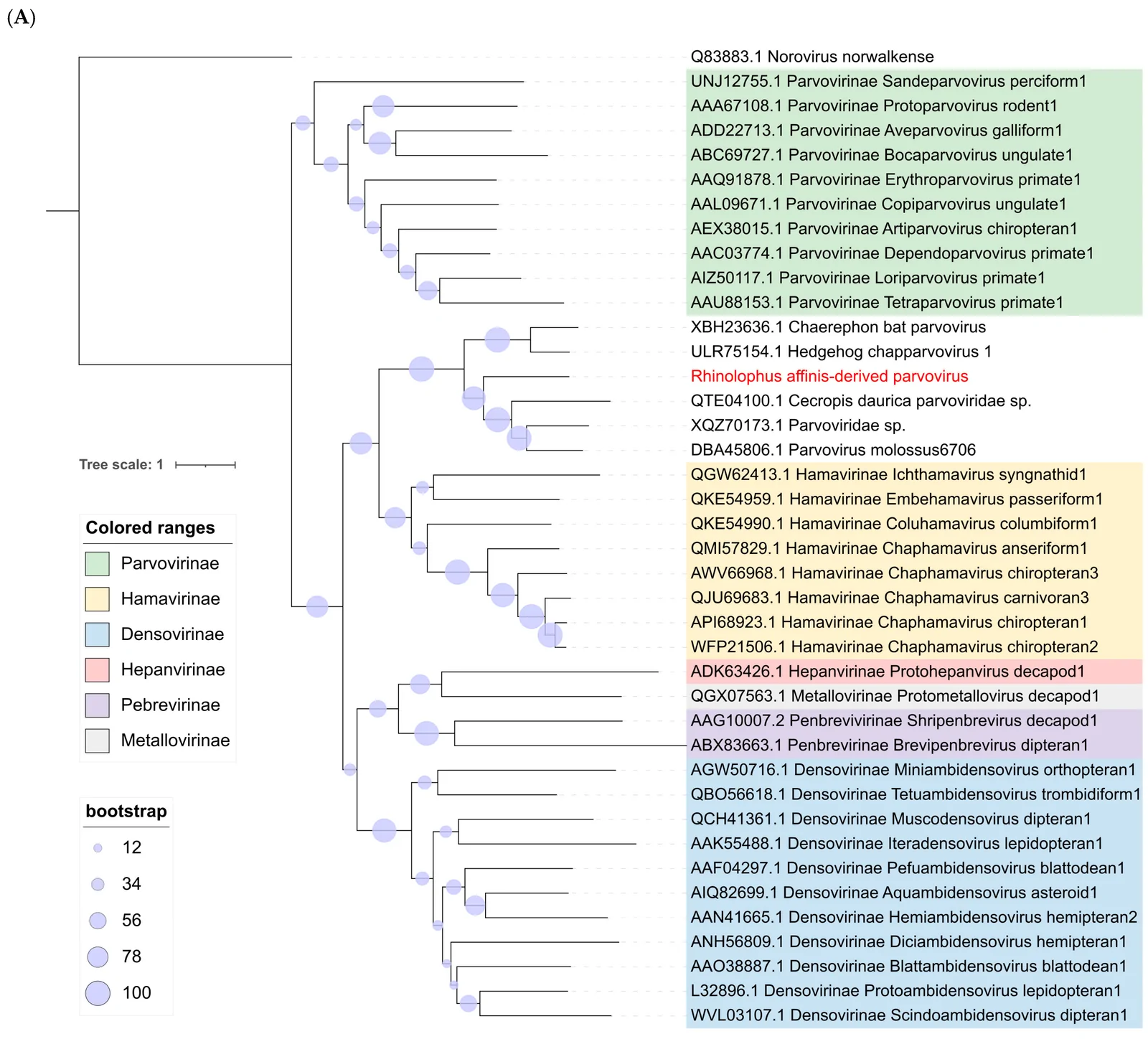

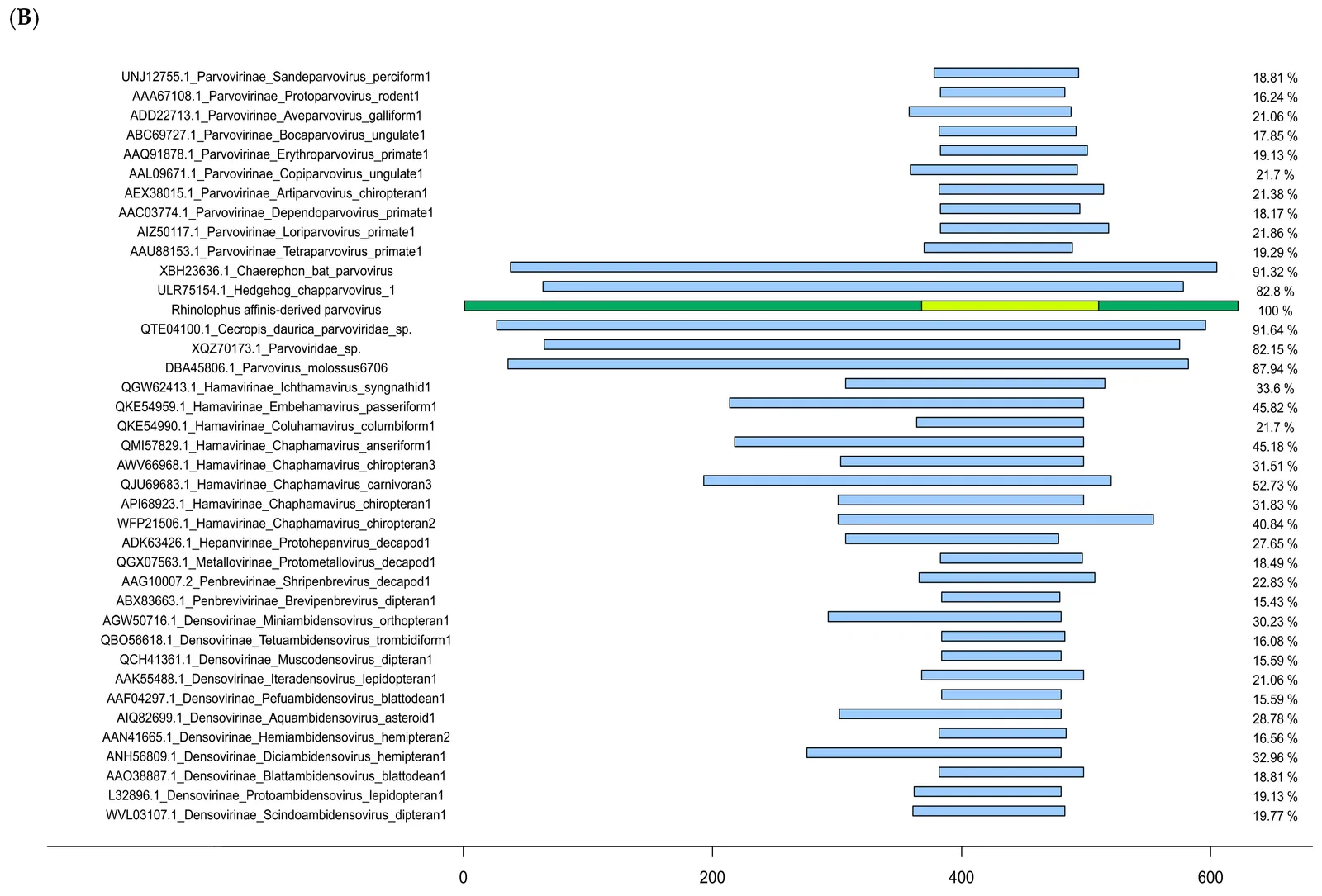
Phylogenetic placement of the *Rhinolophus affinis-associated parvovirus*. (**A**) Phylogenetic tree of the *Parvoviridae* family based on the conserved NS1 helicase domain. Different subfamilies are color-coded, and the novel sequence is highlighted in red. (**B**) Sequence similarity and coverage of NS1 proteins between the novel parvovirus and representative Parvoviridae sequences. The yellow bar indicates the SF3 helicase domain. Regions with >35% identity are colored in green; regions with ≤35% identity are colored in blue.

Homology analysis indicated that the identified virus is most closely related to members of the genus *Ichtchaphamaparvovirus* (Figure 8), with an NS1 amino acid identity (AAI) of 26.5% to the only recognized species in this genus, *Ichthamavirus syngnathid1* (coverage: 49.65%). However, the NS1 protein also showed similar AAI values of 26.5–27.3% to established references from the *Chaphamavirus* genus, including *Chaphamavirus_carnivoran3* (26.18%, coverage: 52.73%), *Embehamavirus passeriform1* (27.06%, coverage 45.82%), and *Chaphamavirus_anseriform1* (23.42%, coverage 45.82%). All these values fall below the genus-level demarcation threshold for the family *Parvoviridae*, which requires members of the same genus to form a monophyletic clade and share more than 35% NS1 AAI with more than 80% coverage [1].

BLAST analysis against the NCBI GenBank database revealed several unclassified ICTV hits sharing homology with the novel virus: QTE04100.1 *Cecropis daurica* parvoviridae sp. (26.91% AAI, 92% coverage), DBA45806.1 *Parvovirus molossus6706* (29.48%, 88%), XQZ70173.1 *Parvoviridae* sp. (32.02%, 82%), ULR75154.1 *Hedgehog chapparvovirus 1* (29.10%, 83%), and XBH23636.1 *Chaerephon bat parvovirus* (28.93%, 91%).

Phylogenetic analysis of the NS1 helicase domain (Figure 8A) placed the identified virus alongside these homologues within a well-supported clade that is sister to the subfamily *Hamaparvovirinae*, with all hamaparvovirus representatives forming an adjacent clade. Consequently, the virus occupies a distinct sister lineage to the clades containing all established *Hamaparvovirinae* genera, suggesting that it may represent a novel taxonomic lineage at the subfamily level.

### 3.9. Analysis of Viral Diversity by Region and Host Trophic Specialization

To study the ecological drivers of virome composition, a comparative analysis of the detected parvoviruses was conducted, incorporating two key parameters: geographic region (Gia Lai and Dong Nai provinces) and host bat diet (insectivore, phytophage, and carnivore). Parvovirus infection was identified in bats from six genera spanning all three trophic strategies: insectivores (*Hipposideros*, *Rhinolophus*, *Murina*, *Pipistrellus*, and *Harpiocephalus*); phytophages (*Cynopterus* and *Rousettus*); and carnivores (*Lyroderma* and *Megaderma*).

**Figure 9:**
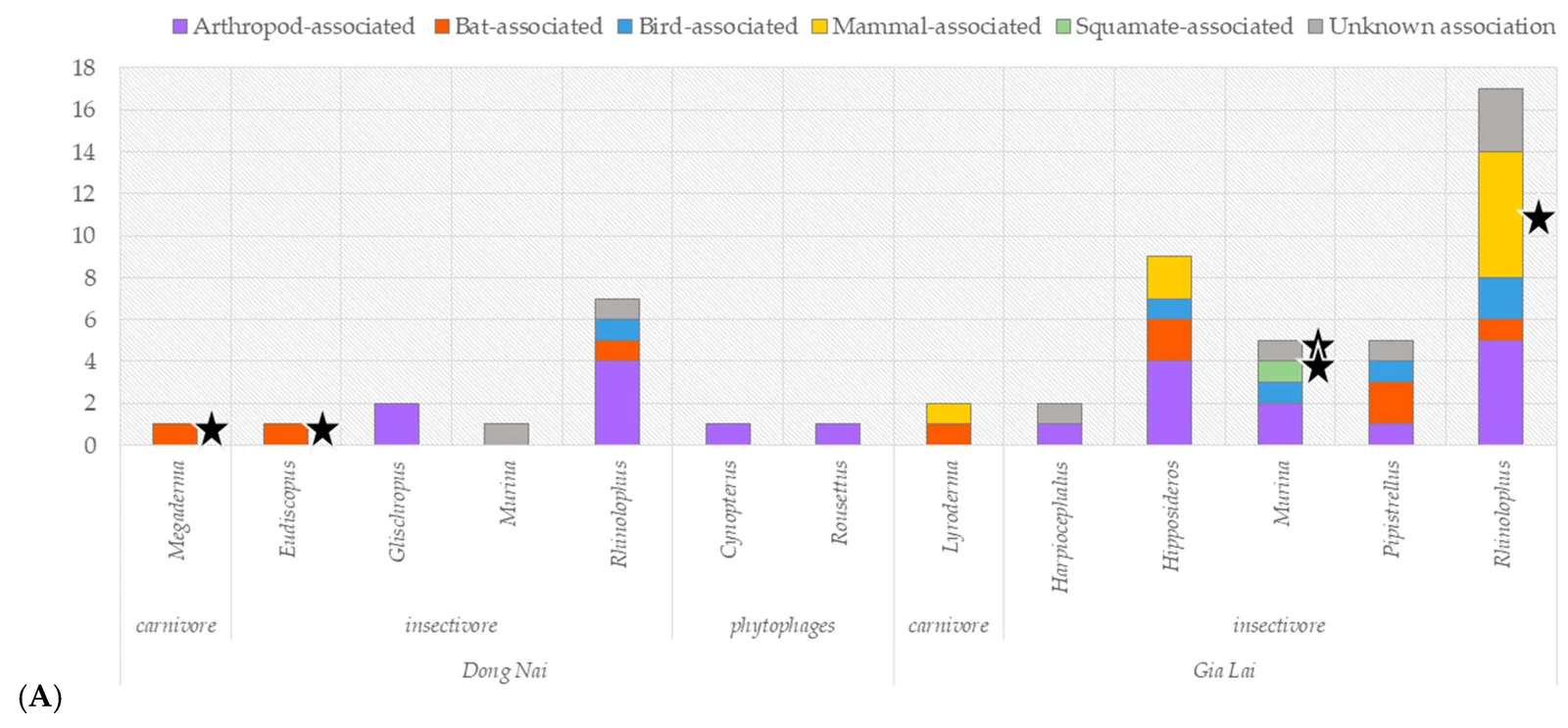

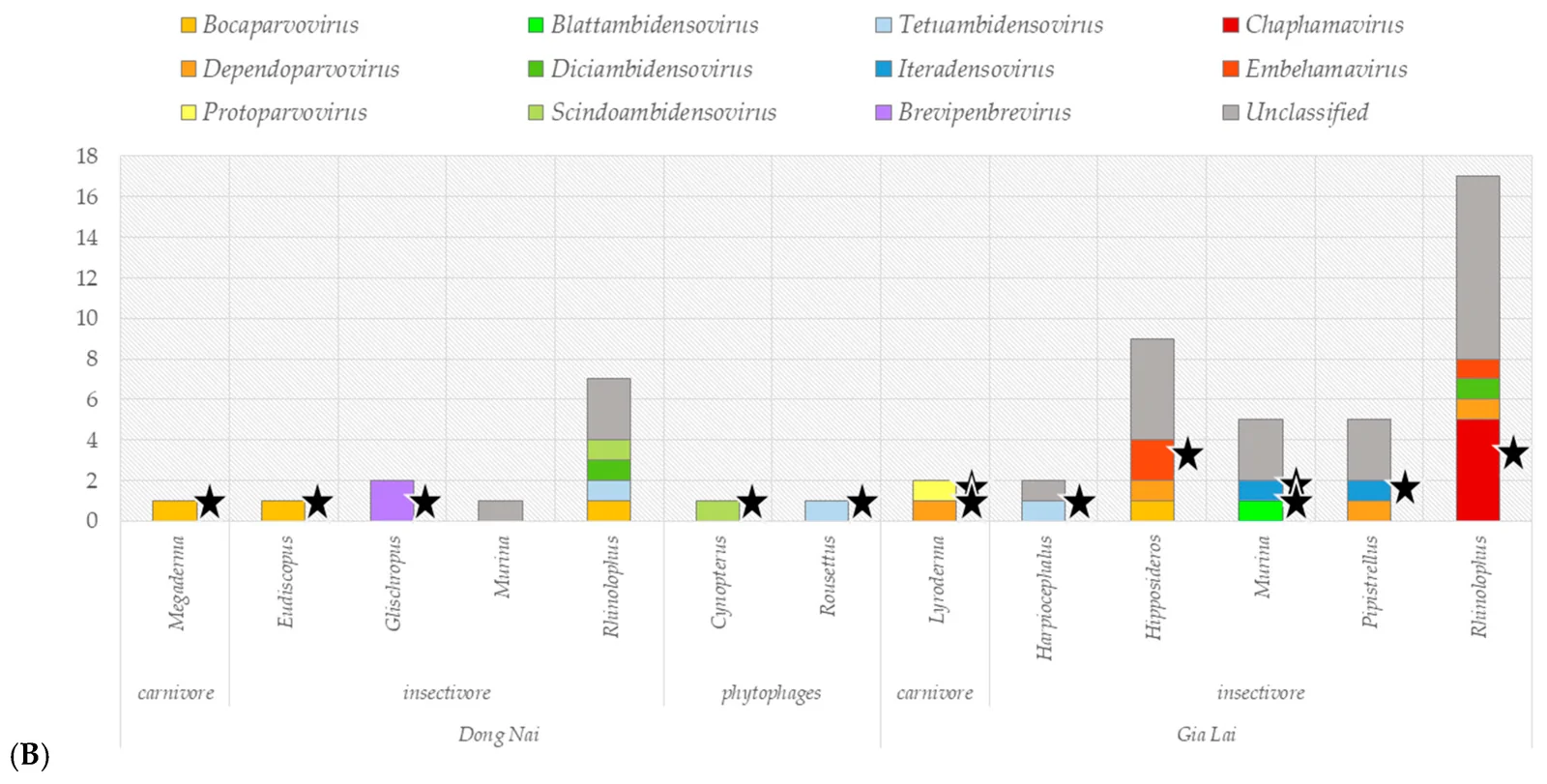
Diversity and distribution of parvoviruses in Vietnamese bats, stratified by host ecology. The vertical axis displays the number of viral sequences detected per bat genus. The horizontal axis organizes the bat genera first by their primary dietary specialization (phytophage, carnivore, and insectivore) and second by the province of collection (Dong Nai and Gia Lai). Viruses showing a significantly higher frequency of association with a specific host genus (AR > 1.96) are indicated with a (*) symbol. (**A**) Stacked bars show the taxonomic composition of viral clusters within each host genus, color-coded by the most probable associated host group for the viruses. (**B**) Stacked bars illustrate the distribution of different detected *Parvoviridae* genera (e.g., *Bocaparvovirus* and *Protoparvovirus*) within each bat host.

A combined visualization (Figure 9) illustrates the distribution of viral taxa across host geographic and trophic categories. Figure 9A features trophic association clusters that depict the composition of viral groups associated with each host species, with inferred trophic links based on established host–virus relationships. Trophic association was assigned based on alignment results against a reference database: the nearest ICTV-classified virus was identified, and its corresponding trophic link was transferred to the sequence obtained in this study. For members of *Densovirinae*, “Arthropod-associated” was assigned. For unclassified *Parvoviridae*, where a definitive link cannot be established, the trophic association is denoted as “unknown”. This visualization facilitates an integrated analysis of the ecological determinants shaping parvovirus diversity within the studied bat populations.

A total of six host-associated groups were identified: arthropod-, bird-, bat-, mammal-, and squamate-associated, and unknown associations. Insect-associated viruses exhibited the greatest diversity, which aligns with the predominantly insectivorous diet of the studied bat species. The bird-associated virus clusters likely reflect overlapping trophic niches, where insectivorous bats and avian share arthropod prey, facilitating potential pathogen exchange through the food chain. Alternatively, there is a possibility of incorrect host determination in the public genomic databases, where the arthropod virus may be incorrectly labeled as a bird one.

A similar visualization (Figure 9B) illustrates the distribution of viral genera across host geographic and trophic categories. Comparison of viral diversity across regions reveals marked geographic differences. In Gia Lai Province, a significantly higher diversity of *Parvoviridae* taxa was shown. This increased taxonomic richness was accompanied by a greater proportion of viral-positive individuals, with a prevalence of 41.3% (26 out of 63). Insectivorous bats predominated in this sample (55 out of 63 individuals). The high viral diversity in Gia Lai was primarily driven by two genera: *Rhinolophus* (13 infected out of 34 examined) exhibited both a high infection rate and a broad range of viral clusters, while *Hipposideros* also contributed significant parvovirus diversity. In contrast, Dong Nai Province yielded only six viral genera (including unclassified) despite a comparable number of insectivorous bats examined (40 individuals).

The genus *Murina* displayed distinct viral compositions between regions: in Gia Lai, viral positive individuals (two of five studied) harbored four different groups associated with bats, and arthropods and belonged to three genera (including unclassified), while in Dong Nai, only a single “unknown association” group was detected in one infected individual (one of six studied), unclassified genus. It is important to note that different *Murina* species were represented in each region. The genus *Rhinolophus* showed the highest parvovirus positivity rate (over 35% at each site) and the broadest viral taxa divergence.

Collectively, these findings highlight the high genetic diversity of parvoviruses circulating in the studied bat populations and underscore the complex composition of their viral pool. This assemblage includes both host-specialized viral lineages and those whose presence appears linked to ecological and dietary factors, reflecting the multifaceted interactions shaping the bat virome. The observed inter-regional differences are attributable not to the total number of animals sampled but rather to the distinct taxonomic composition of the local bat communities. In both Gia Lai and Dong Nai, *Rhinolophus* bats were the primary contributors to viral diversity. In Gia Lai, however, *Hipposideros* also emerged as a key contributor, together with *Rhinolophus* driving the heightened diversity observed in that province.

## 4. Discussion

The results demonstrate that Vietnam harbors substantial parvovirus diversity, supporting a broad range of circulating members within the family Parvoviridae. This aligns with the established concept of bats playing a key role in the maintenance and generation of viral diversity [52]. The high species diversity of Vietnam’s bat fauna [21] creates favorable conditions for the circulation of diverse viruses, including rare and previously uncharacterized taxa [53].

High bat species diversity in the region was confirmed, with a total of 26 species collected across two provinces over a limited sampling period. In Dong Nai Province, *C. sphinx* was the dominant species. This prevalence reflects anthropogenic influence within Bu Gia Map National Park. In Gia Lai Province, the dominant species was *Rh. affinis*. This insectivorous bat is widespread across Southeast Asia and is characteristic of Vietnam’s mountainous regions [21]. Notably, the sampling in Gia Lai confirmed the presence of *M. beelzebub*, a species previously documented only through isolated encounters in Central Vietnam [20]. Furthermore, the presence of the rare *Eudiscopus denticulus* was confirmed in Bu Gia Map National Park (Dong Nai Province) [21,54].

This remarkable bat diversity provides a rich substrate for viral evolution. This study revealed a substantial diversity of parvoviruses. A significant fraction of the discovered sequences lacked sufficient similarity for definitive classification at the species level, underscoring the presence of numerous previously unrecognized OTUs in the studied region. This high proportion of potentially novel parvoviruses is consistent with prior studies on East Asian bats, which have noted substantial diversity within this family [26,55].

Of particular interest are the sequences tentatively assigned to a bat-associated *Parvovirinae* group, which were identified primarily in Gia Lai province. Given that this subfamily infects vertebrates, including a broad range of mammals [1], their detection in bats is highly relevant. Most identified sequences exhibit low similarity to their closest known relatives, indicating a high degree of genetic divergence and the presence of novel viral lineages.

The bocaparvoviruses and protoparvoviruses identified in this study fall well within the established taxonomy of their respective genera, forming a common clade with other chiropteran *Parvoviridae* members [56]. However, the majority of the detected sequences fall below the species demarcation threshold, thus representing new OTUs within these genera.

The detection of dependoparvoviruses in various bat species in our study further confirms the global circulation of this genus in bat populations [25,57,58] and further expands our understanding of chiropteran dependoparvovirus diversity. While two of the observed sequences are either classified as known ICTV accepted species, two other sequences, each forming a distinct OTU, were related to dependoparvoviruses infecting primates and pinnipeds, although with low amino acid identity. The detection of four genetically distinct dependoparvoviruses at a single location, despite the limited sample size, suggests that the diversity of this genus in bats is substantially underestimated. This finding aligns with targeted studies that have reported high genetic diversity and multiple divergent genotypes within bat populations [25]. Additionally, given the dependence of dependoparvoviruses on helper viruses, their presence indirectly indicates the circulation of as-yet-unidentified adenoviruses or herpesviruses in the studied populations [53].

Of particular note is the subfamily *Hamavirinae* [3,31]. In this study, we detected sequences belonging to two genera within this subfamily: *Chaphamavirus* and *Embehamavirus*. The chaphamavirus sequences identified in several *Rh. affinis* samples showed homology to *Chaphamavirus carnivoran1*, a virus associated with enteric disease in dogs [14]. The detection of genetically close variants in several individuals of the same bat species suggests intraspecific circulation within the population. Moreover, the degree of genetic divergence from known sequences provides a basis for considering the detected variants as belonging to the potentially novel species. Notably, although chaphamaviruses sequences have previously been reported in bats from other regions [18,59], our analysis revealed no significant similarity between our sequences and *Chaphamavirus chiropteran*. This finding suggests a potential role of *Rh. affinis* as a natural reservoir for this novel viral group, though its biological significance in bat hosts remains unclear.

Three additional OTUs classified as likely belonging to the genus *Embehamavirus* were detected in *Rh. affinis* and *Hip. gentilis*. Together with our viral sequences, this clade includes a parvovirus sequence previously recovered from the blood plasma of a patient with neuroinfection in Brazil [60]. Although this virus has not yet received official taxonomic status, the genetic similarity between the *Rh. affinis* isolate and this human-derived sequence substantially exceeds the species-level demarcation threshold. The detection of closely related viruses in a bat host and in the plasma virome of a febrile Brazilian patient raises important questions about the potential zoonotic origin of this viral lineage. However, we acknowledge that the original study does not provide definitive evidence of active viral replication in the human patient, and alternative interpretations must be considered. Thus, we interpret the genetic similarity as an indicator of close evolutionary relationship that warrants further investigation, rather than as conclusive evidence of zoonotic transmission. Further research is necessary to clarify the evolutionary relationships within this cluster and to assess its role in human pathology.

In addition to viruses clearly attributable to specific genera and subfamilies, metagenomic analysis of a sample from *Rh. affinis* enabled the whole genome characterization of a previously undescribed virus within the family *Parvoviridae*. The level of genetic divergence between this virus and all known subfamily representatives falls below ICTV thresholds for genus assignment. This suggests that it may represent not only a novel species but also a candidate for a novel taxonomic group at the subfamily level. The phylogenetic clustering of our novel sequence with viruses detected from diverse hosts and sample types, including a bird cloacal metagenome (QTE04100.1) [50], a free-tailed bat saliva metagenome (DBA45806.1) [61], a blackfly metagenome (XQZ70173.1) [62], an Amur hedgehog (ULR75154.1), and another bat metagenome (XBH23636.1) [63], provides intriguing insights into the potential host range of this clade. The presence of a virus sequence in an arthropod metagenome (blackfly), together with its close genetic relationship to viruses detected in vertebrates (birds, bats, and hedgehog), raises questions about the true natural reservoir of these viruses. Notably, the mammalian hosts from which these related sequences were obtained, including bats and hedgehogs, are all insectivorous. This raises the possibility that the virus was acquired through dietary exposure to infected arthropods and that the detected mammals may not represent the true natural reservoir. Alternatively, the virus may circulate among a broad range of mammals, with the blackfly representing either a contaminated sample or a genuine component of a vector-borne transmission cycle. Given the absence of definitive host association data, these findings underscore the need for targeted screening of potential reservoir and vector species to establish the true host range of this viral lineage. Nevertheless, this finding aligns with recent studies highlighting the rapidly expanding known diversity of parvoviruses [15,31] and underscores that the taxonomy of the family *Parvoviridae* remains incomplete, requiring ongoing revision as new genomic data emerge.

Regarding the unclassified *Parvoviridae* viruses identified in this study, their phylogenetic placement and host associations remain ambiguous. Although their detection in bat feces is consistent with bats serving as the true hosts, their genomic similarity to viruses from ecologically disparate sources, such as birds, makes it impossible to determine whether they infect bats directly or are associated with the bats’ diet or habitat. This ambiguity is further supported by the frequent detection of *Densovirinae* (invertebrate-associated parvoviruses) representatives, which likely reflects the bats’ insectivorous diet. Consequently, whether these viruses are specific to chiropteran hosts or are ecologically linked through prey or environmental exposure cannot be determined from the present data.

Several additional limitations warrant consideration. First, the apparent number of novel taxa may be inflated, as distinct OTUs could correspond to different genomic regions of the same virus in cases where a complete genome sequence was not obtained. Second, the absence of full genome sequences in most cases precluded definitive ICTV-based classification, thereby constraining the resolution of our taxonomic assignments. Although taxonomic assignments remain provisional, the detected sequences provide insights into parvovirus diversity and evolutionary relationships of bat-associated parvoviruses in Vietnam.

Within DNA viruses, parvoviruses exhibit a relatively high mutation rate and documented instances of host switching [12,64], while also being considered to generally co-evolve with their hosts [65]. This study uncovered a substantial, previously unrecognized diversity of parvoviruses in the investigated Vietnamese provinces, with the majority of detected variants representing potentially novel genetic lineages. These findings enhance the understanding of parvovirus diversity and evolution in Southeast Asia and provide a foundation for assessing potential zoonotic risks [23,24,53].

Additionally, host dietary specialization and local population structure strongly shape viral assemblages. In particular, insectivorous bats from the genera *Rhinolophus* and *Hipposideros* in Gia Lai Province were central to supporting viral diversity. A more restricted viral profile was found in Dong Nai Province despite a similar sampling effort focused on insectivorous hosts.

Collectively, these outcomes highlight the necessity of a comprehensive approach to investigating the bat virome, both globally and in specific ecosystems like those in Vietnam. They establish a basis for deciphering the ecological and evolutionary drivers of virus circulation and diversification within these natural reservoirs. The research also provides important groundwork for evaluating and anticipating potential risks related to zoonotic transmission.

## Figures and Tables

**Table 1:** Composition of the primer pool.

Primer ID	Primer Sequence, 5′ → 3′
bats_1020312_fw	NWCAMCACAGGHCTGGCA
bats_336066_fw	CRCWTCYCWCCACAAGAY
bats_752876_fw	KGGCCHHTAATHTGTGCW
bats_887125_rv	NACCCANCCRCADTCAAA
bats_889523_rv	DSCVGCTGTNAGGTCRTT
bats_909740_rv	GTTGGTTCTCCTTGGTCW
bats_911390_fw	ACCTGAACATACACAACC
bats_fw_Bocoparvovirus_fw	TTTRTHTTYAAYGAYTGC
bats_Bocoparvovirus_2_rw	YAWAGKYCTGTGAATGAGA

**Table 2:** Genome organization of the *Rhinolophus affinis*-derived parvovirus.

Protein	Start	End	Frame	Sense	Length
Nonstructural protein (NS1)	2559	4428	3	+	1869
Capsid protein (VP)	4430	6008	2	+	1578
Hypothetical protein1	56	433	2	+	378
Hypothetical protein2	433	1396	1	+	963
Hypothetical protein3	1388	1675	2	+	287
Hypothetical protein4	1754	1978	2	+	224
Hypothetical protein5	2089	2532	1	+	443

## Data Availability

Genomic sequences are deposited in GenBank under accession numbers PZ507310–PZ507363.

## Supplementary Materials

The following supporting information can be downloaded at https://www.mdpi.com/article/10.3390/v18080860/s1, Table S1: Sample metadata and parvovirus detection. Table S2: Taxonomic assignment and identity scores of parvoviral sequences. Table S3: Reference database composition for primer design. Figure S1: Tanglegram comparing VP1 and NS1 phylogenies of *Bocaparvovirus*. Figure S2: Tanglegram comparing VP1 and NS1 phylogenies of *Protoparvovirus*. Figure S3: Tanglegram comparing VP1 and SF3 phylogenies of *Hamaparvovirinae*.
